# Abnormal serum chloride is associated with increased mortality among unselected cardiac intensive care unit patients

**DOI:** 10.1371/journal.pone.0250292

**Published:** 2021-04-26

**Authors:** Thomas J. Breen, Benjamin Brueske, Mandeep S. Sidhu, Kianoush B. Kashani, Nandan S. Anavekar, Gregory W. Barsness, Jacob C. Jentzer

**Affiliations:** 1 Department of Internal Medicine, Mayo Clinic, Rochester MN, United States of America; 2 Division of Cardiology, Department of Medicine, Albany Medical Center and Albany Medical College, Albany, NY, United States of America; 3 Division of Nephrology & Hypertension, Department of Internal Medicine, Mayo Clinic, Rochester, MN, United States of America; 4 Division of Pulmonary and Critical Care Medicine, Department of Internal Medicine, Mayo Clinic, Rochester, MN, United States of America; 5 Department of Cardiovascular Medicine, Mayo Clinic, Rochester, MN, United States of America; Erasmus Medical Centre: Erasmus MC, NETHERLANDS

## Abstract

**Purpose:**

We sought to describe the association between serum chloride levels and mortality among unselected cardiac intensive care unit (CICU) patients.

**Materials and methods:**

We retrospectively reviewed adult patients admitted to our CICU from 2007 to 2015. The association of dyschloremia and hospital mortality was assessed in a multiple variable model including additional confounders, and the association of dyschloremia and post-discharge mortality were assessed using Cox proportional-hazards analysis.

**Results:**

9,426 patients with a mean age of 67±15 years (37% females) were included. Admission hypochloremia was present in 1,384 (15%) patients, and hyperchloremia was present in 1,606 (17%) patients. There was a U-shaped relationship between admission chloride and unadjusted hospital mortality, with increased hospital mortality among patients with hypochloremia (unadjusted OR 3.0, 95% CI 2.5–3.6, p<0.001) or hyperchloremia (unadjusted OR 1.9, 95% CI 1.6–2.3, p<0.001). After multivariate adjustment, hypochloremia remained associated with higher hospital mortality (adjusted OR 2.1, 95% CI 1.6–2.9, p <0.001). Post-discharge mortality among hospital survivors was higher among patients with admission hypochloremia (adjusted HR 1.3, 95% CI 1.1–1.6; p<0.001).

**Conclusion:**

Abnormal serum chloride on admission to the CICU is associated with increased short- and long-term mortality, with hypochloremia being a strong independent predictor.

## Introduction

Serum chloride abnormalities are common among hospitalized patients, and both hypochloremia and hyperchloremia have been associated with increased in-hospital mortality among general intensive care unit (ICU) patients [1–4]. Hypochloremia is an independent marker of short- and long-term mortality among patients with heart failure (HF) and predicts a decreased response to diuretics [5–8]. The finding that serum chloride derangements are potentially associated with adverse outcomes among ICU and cardiovascular disease populations reflects the importance of serum chloride in normal physiology.

Serum chloride is influenced by numerous pathophysiologic processes and plays a key role in the maintenance of osmotic pressure, acid-base disturbances, and regulation of renal function [9]. Serum chloride and sodium levels correlate closely to maintain plasma electroneutrality, and changes in volume status and plasma tonicity typically produce parallel changes in serum sodium and chloride levels. Unlike serum sodium, chloride levels are also intimately associated with acid-base status, with hyperchloremia typically associated with non-anion gap metabolic acidosis and hypochloremia typically associated with metabolic alkalosis [10–12]. Anion-gap acidosis may be associated with relative hypochloremia and has been linked to adverse outcomes among CICU patients [13]. Prior studies assessing the associations between serum chloride and patient outcomes are limited by the lack of information regarding sodium, anion gap, and acid-base status.

The modern cardiac intensive care unit (CICU) cares for a heterogeneous population of critically ill patients with concomitant cardiovascular disease, yet there are no published studies examining the significance of abnormal serum chloride levels among CICU patients. The aim of our study was to clarify whether an abnormal admission chloride level was associated with higher hospital and post-discharge mortality among CICU patients and to provide insights about the effects of associated electrolyte and acid-base disturbances.

## Methods

### Participants

The Mayo Clinic Institutional Review Board approved this historical cohort study as a minimal risk study that was exempt from informed consent. We analyzed a database of adult Mayo Clinic CICU (Rochester, MN) patients ≥18 years old whose admission fell entirely between January 1, 2007, and December 31, 2015 and consented to have their medical records used for research under Minnesota state law statute 144.295 [14–17]. The Mayo Clinic CICU cares for medically critically ill patients with cardiovascular disease, not including post-cardiotomy patients or patients receiving extracorporeal membrane oxygenator support. Patients were identified from archived electronic health records, and only the data from the first CICU admission was included to avoid survival bias associated with readmissions [18]. We excluded patients without available data on admission chloride or creatinine values.

### Collected data

Demographics, vital signs, laboratory results, diagnoses, procedures, therapies and length of stay (LOS) were extracted from the electronic medical record (EMR) through the Multidisciplinary Epidemiology and Translational Research in Intensive Care Data Mart [18, 19]. Admission diagnoses were defined as all International Classification of Diseases, Ninth Revision (ICD-9) diagnosis codes recorded within one day before or after CICU admission [20, 21]. EMR data during the first 24 hours of CICU admission were used to automatically calculate the Sequential Organ Failure Assessment (SOFA) and Acute Physiology and Chronic Health Evaluation (APACHE)-III scores and APACHE-IV predicted mortality via imputation of missing variables as normal [15–17, 22–24]. SOFA scores were calculated daily on each CICU day, and the mean of all daily SOFA scores was calculated. The Charlson Comorbidity Index and baseline comorbidities were determined using a previously-validated electronic algorithm [25]. All-cause CICU, in-hospital, and post-discharge mortality were determined using notification of patient death in the electronic medical record as of February 1, 2018 [14–17, 26].

### Definitions

We defined admission chloride as the chloride value closest to the time of CICU admission. Serum chloride levels were the default, but plasma chloride values were used if serum chloride was not available. Patients were grouped based on the normal reference range for admission chloride levels: normal admission chloride (admission chloride 98–107 mEq/L), admission hypochloremia (admission chloride <98 mEq/L) and admission hyperchloremia (admission chloride ≥108 mEq/L). The maximum and minimum chloride levels during the CICU admission were collected, and patients were grouped as follows: normal chloride (minimum chloride ≥98 mEq/L and maximum chloride <108 mEq/L), hypochloremia (minimum chloride <98 mEq/L) and hyperchloremia (maximum chloride ≥108 mEq/L). Hyponatremia was defined as admission sodium level <135 mEq/L, and hypernatremia was defined as an admission sodium level ≥145 mEq/L [27]. The admission anion gap was defined as (admission sodium level–chloride level–bicarbonate level) using values from a single serum or plasma sample, with an anion gap >12 mEq/L considered elevated. Metabolic acidosis was defined as admission bicarbonate level <22 mEq/L, and metabolic alkalosis was defined as admission bicarbonate level >26 mEq/L. Among patients who had not previously received dialysis, we defined the highest AKI stage during hospitalization based on modified KDIGO criteria; mild AKI was defined as stage 1 AKI, and severe AKI was defined as stage 2 or 3 AKI [21, 28].

### Statistical analysis

Hospital mortality was the primary enpoint. Secondary endpoints included CICU mortality and mortality in the initial five years after discharge among hospital survivors. Continuous variables were compared using analysis of variance (ANOVA), while categorical variables were compared using Pearson chi-square tests. Logistic regression was used to calculate odds ratios (OR) and 95% confidence intervals (CI). Multivariable analysis was performed to identify predictors of hospital mortality using a non-adaptive elastic net penalized regression model for variable selection, with candidate variables including demographics, comorbidities, illness severity, admission laboratory values, admission diagnoses, and CICU therapies and complications [29]. Ideal tuning parameters for the elastic net were selected by 150-point linear grid search with 10-fold cross-validation to maximize the area under the curve (AUC) [30]. Kaplan-Meier analysis was used to determine post-discharge survival in hospital survivors, with groups compared using the log-rank test. Hazard ratio (HR) and 95% CI values for post-discharge mortality were determined using Cox proportional-hazards analysis, after adjusting for predictors of in-hospital mortality from the logistic regression model. Two-tailed P values <0.05 were considered statistically significant. Analyses were performed using JMP Pro 14.1.0 (SAS Institute, Cary NC).

## Results

### Study population

12,904 CICU admissions were screened and 2,900 met the initial exclusion criteria for the study cohort, as previously described [15, 17, 22, 27]. Of the remaining 10,004 patients, we excluded an additional 456 (4.6%) patients without an admission chloride level and 122 (1.2%) patients without an available creatinine level, yielding a final study population of 9,426 unique CICU patients (**S1 Fig**). The mean age of included patients was 67±15 years with 3,519 (37%) females. The baseline characteristics of the final study population are listed in **Table 1**. The mean admission chloride value was 102.8±5.5 mEq/L, with a distribution shown in **S2 Fig**.

**Table 1:** Baseline characteristics of patients with normal chloride (admission chloride 98–107 mEq/L), hypochloremia (admission chloride <98 mEq/L) and hyperchloremia (admission chloride ≥108 mEq/L). Data displayed as number (%) for categorical variables and mean ± standard deviation for continuous variables. Groups were compared using ANOVA for continuous variables and chi-square tests for categorical variables.

Variable	Normal chloride (n = 6436)	Hypochloremia (n = 1384)	Hyperchloremia (n = 1606)	P value
***Demographics***				
Age	67.4±15.1	67.1±15.2	67.8±15.4	0.41
Female gender	2277 (35.4%)	585 (42.3%)	657 (40.9%)	<0.001
White race	5990 (93.1%)	1248 (90.2%)	1477 (92.0%)	<0.001
BMI	29.6±6.9	30.3±8.1	28.5±6.3	<0.001
***Comorbidities***				
Charlson Comorbidity Index	2.2±2.5	3.5±2.8	2.1±2.6	<0.001
Prior myocardial infarction	1282 (20.0%)	278 (20.2%)	315 (19.6%)	0.92
Prior heart failure	1118 (17.4%)	541 (39.3%)	200 (12.4%)	<0.001
Prior stroke	790 (12.3%)	193 (14.0%)	181 (11.3%)	0.07
Prior diabetes mellitus	1775 (27.6%)	557 (40.4%)	366 (22.8%)	<0.001
Prior cancer	1319 (20.6%)	349 (25.3%)	335 (20.9%)	<0.001
Prior lung disease	1196 (18.6%)	381 (27.7%)	240 (14.9%)	<0.001
Prior CKD	1108 (17.3%)	516 (37.5%)	303 (18.9%)	<0.001
Prior dialysis	254 (4.0%)	231 (16.7%)	62 (3.9%)	<0.001
***Admission diagnoses***				
Acute coronary syndrome	2926 (46.0%)	378 (27.5%)	723 (45.6%)	<0.001
Heart failure	2794 (43.9%)	1035 (75.2%)	549 (34.6%)	<0.001
Cardiac arrest	685 (10.8%)	148 (10.8%)	276 (17.4%)	<0.001
Shock	717 (11.3%)	242 (17.6%)	298 (18.8%)	<0.001
Cardiogenic shock	568 (8.9%)	197 (14.3%)	233 (14.7%)	<0.001
Sepsis	345 (5.4%)	131 (9.5%)	107 (6.7%)	<0.001
Respiratory failure	1164 (18.3%)	420 (30.5%)	392 (24.7%)	<0.001
***Severity of illness***				
APACHE-III score	59.1±24.1	69.8±22.5	66.6±29.2	<0.001
APACHE-IV mortality	0.154±0.188	0.219±0.197	0.212±0.242	<0.001
Day 1 SOFA score	3.2±3.0	4.8±3.3	4.3±3.7	<0.001
Mean week 1 SOFA	2.7±2.4	4.1±2.8	3.6±2.1	<0.001
***Acute Kidney Injury***				
Any AKI	2911 (47.1%)	882 (76.5%)	785 (50.8%)	<0.001
No AKI	3405 (52.9%)	325 (23.5%)	790 (49.2%)	<0.001
KDIGO stage 1	2153 (33.5%)	559 (40.4%)	567 (35.3%)	<0.001
KDIGO stage 2	507 (7.9%)	235 (17.0%)	131 (8.2%)	<0.001
KDIGO stage 3	371 (5.8%)	264 (19.1%)	120 (17.5%)	<0.001
***Therapies and procedures***				
Invasive ventilator	902 (14.0%)	211 (15.2%)	424 (26.4%)	<0.001
Noninvasive ventilator	900 (14.0%)	374 (27.0%)	192 (12.0%)	<0.001
Vasopressors	1096 (17.0%)	413 (29.8%)	430 (26.8%)	<0.001
Inotropes	487 (7.6%)	288 (20.8%)	100 (6.2%)	<0.001
Coronary angiography	3580 (55.6%)	536 (38.7%)	851 (53.0%)	<0.001
PCI	2399 (37.3%)	277 (20.0%)	608 (37.9%)	<0.001
IABP	533 (8.3%)	131 (9.5%)	169 (10.5%)	0.01
Pulmonary artery catheter	419 (6.5%)	196 (14.2%)	96 (6.0%)	<0.001
RBC transfusion	666 (10.4%)	186 (13.4%)	296 (18.4%)	<0.001
Dialysis	223 (3.5%)	173 (12.5%)	66 (4.1%)	<0.001
CRRT	66 (1.0%)	79 (5.7%)	22 (1.4%)	<0.001
***Admission laboratory data***				
Sodium (mEq/L)	138.1±3.3	132.6±5.4	141.0±3.4	<0.001
Hypernatremia	774 (12.0%)	4 (0.3%)	36 (2.2%)	<0.001
Hyponatremia	108 (1.7%)	847 (61.2%)	193 (12.0%)	<0.001
Potassium (mEq/L)	4.3±0.6	4.4±0.8	4.2±0.7	<0.001
Bicarbonate (mEq/L)	24.1±3.7	26.2±5.7	21.3±3.9	<0.001
Metabolic acidosis	1382 (21.5%)	255 (18.4%)	792 (49.3%)	<0.001
Metabolic alkalosis	1501 (23.3%)	625 (45.2%)	92 (5.7%)	<0.001
Chloride (mEq/L)	103.0±2.6	93.5±3.8	110.2±2.7	<0.001
Anion gap (mEq/L)	11.0±3.8	12.9±4.8	9.6±4.2	<0.001
Elevated anion gap	1984 (30.8%)	715 (51.7%)	323 (20.1%)	<0.001
BUN (mg/dL)	24.2±15.9	39.1±26.5	25.4±17.2	<0.001
Creatinine (mg/dL)	1.2±0.8	2.1±2.0	1.2±0.8	<0.001
***Outcomes***				
CICU mortality	231 (3.6%)	125 (9.0%)	133 (8.3%)	<0.001
Hospital mortality	393 (6.1%)	227 (16.4%)	180 (11.2%)	<0.001
CICU LOS	2.4±4.5	3.6±6.9	2.5±2.5	<0.001
Hospital LOS	7.2±10.4	13.2±23.6	7.0±10.3	<0.001

AKI = acute kidney injury; APACHE = Acute Physiology and Chronic Health Evaluation; BMI = body mass index; BUN = blood urea nitrogen; CICU = cardiac intensive care unit; CKD = chronic kidney disease; CRRT = continuous renal replacement therapy; IABP = intra-aortic balloon pump; KDIGO = kidney disease improving global outcomes; LOS = length of stay; PCI = percutaneous intervention; RBC = red blood cell; SOFA = sequential organ failure assessment.

Admission hypochloremia was present in 1,384 (15%) patients, and admission hyperchloremia was present in 1,606 (17%) patients. Patients with admission hypochloremia or hyperchloremia differed from patients with normal admission chloride in terms of baseline characteristics, including comorbidities, illness severity, admission diagnoses, admission laboratory values and procedures, and therapies (**Table 1**). Patients with admission hypochloremia had a higher prevalence of HF, worse renal function, and greater overall illness severity than patients with admission hyperchloremia or normal admission chloride. Patients with admission hypochloremia had lower admission sodium and higher admission anion gap and bicarbonate, while patients with admission hyperchloremia had higher admission sodium and lower admission bicarbonate. CICU and hospital length of stay were higher in patients with hypochloremia compared with normal chloride or hyperchloremia (p <0.001).

### Admission chloride and hospital mortality

**Fig 1:**
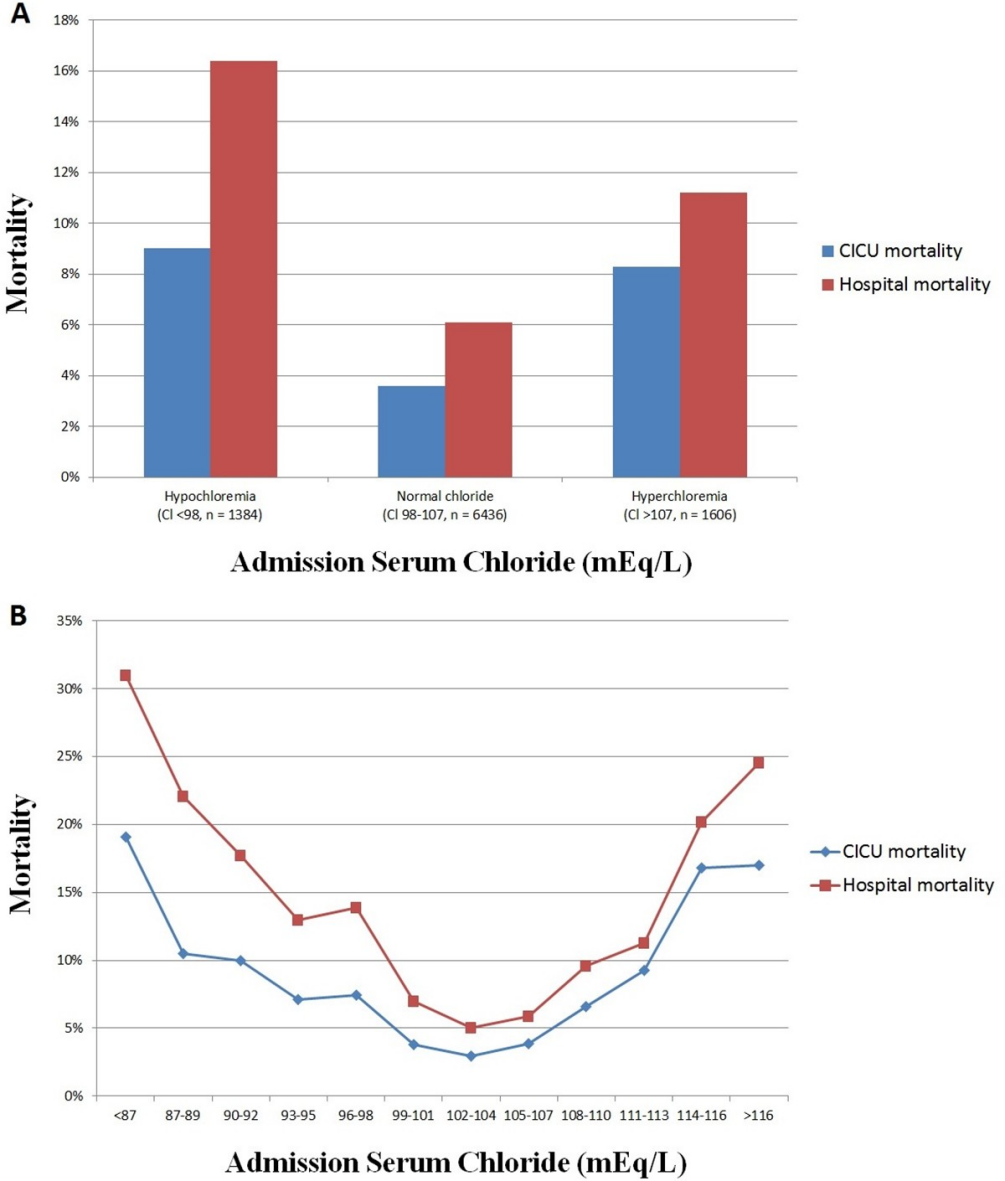
CICU and hospital mortality and admission chloride level in the overall population, based on the presence of admission hypochloremia or hyperchloremia (A) or as a function of admission chloride level (B). Hypochloremia is defined as chloride <98 mEq/L and hyperchloremia is defined as chloride ≥108 mEq/L.

Hospital mortality occurred in 800 (8.5%) patients, including 489 (5.2%) patients who died in the CICU. Compared to patients with a normal admission chloride (**Fig 1A**), an elevated risk of unadjusted hospital mortality was observed in patients with admission hypochloremia or admission hyperchloremia, and patients with admission hypochloremia had higher hospital mortality than patients with admission hyperchloremia (all p <0.001). There was a U-shaped relationship between admission chloride and unadjusted hospital and CICU mortality (**Fig 1B**).

After multivariable adjustment (**Table 2**), a higher admission chloride level remained associated with lower in-hospital mortality (adjusted OR per 1 mEq/L 0.95, 95% OR 0.93–0.98, p = 0.002); the final model validation AUC was 0.937. Those with admission hypochloremia were at elevated risk of in-hospital mortality compared with patients with normal admission chloride (adjusted OR 2.13, 95% CI 1.56–2.91, p <0.001) or admission hyperchloremia (adjusted OR 2.18, 95% CI 1.38–3.44, p <0.001). Patients with admission hyperchloremia were not at increased risk of in-hospital mortality (adjusted OR 0.98, 95% CI 0.71–1.34, p = 0.89).

**Table 2:** Predictors of hospital mortality on multivariable elastic net regression. Only predictors with p <0.1 are shown. Additional predictors included in the model were female gender, white race, BMI, APACHE-III score, prior stroke, prior HF, prior stroke, prior CKD, prior diabetes, prior liver disease, prior lung disease, prior dialysis invasive ventilator, noninvasive ventilator, RBC transfusion, PAC, IABP, coronary angiography, CRRT, number of vasoactive drugs, admission diagnosis of sepsis, admission diagnosis of VT/VF, admission diagnosis of ACS, admission systolic BP, admission HR, admission oxygen saturation, admission respiratory rate, admission MAP, admission GCS, admission hemoglobin, admission sodium, admission potassium, admission bicarbonate. Candidate variables not included in the model were prior cancer, admission diastolic BP, admission WBC count, admission diagnosis of CS, and admission diagnosis of HF. Final model AUC = 0.937.

Variable	Adjusted OR	95% CI	P value
Age (per year)	1.027	1.017–1.038	<0.001
Mean week 1 SOFA score	1.454	1.373–1.539	<0.001
Charlson Comorbidity Index	1.078	1.016–1.142	0.01
PCI	0.724	0.541–0.969	0.03
Dialysis	2.184	1.307–3.649	0.003
*Admission diagnoses*			
Cardiac arrest	4.651	3.426–6.313	<0.001
Shock	1.678	1.198–2.351	0.003
Respiratory failure	1.407	1.033–1.918	0.03
SVT/AF	0.738	0.574–0.950	0.02
*Admission laboratory data*			
Chloride (per mEq/L)	0.954	0.926–0.982	0.002
BUN (per mg/dL)	1.011	1.04–1.017	<0.001
Creatinine (per mg/dL)	0.868	0.764–0.985	0.03
Neutrophils (per 10^3^ per mm^3^)	1.032	1.010–1.055	0.005

ACS = acute coronary syndrome; AF = atrial fibrillation; AKI = acute kidney injury; APACHE = Acute Physiology and Chronic Health Evaluation; BMI = body mass index; BP = blood pressure; BUN = blood urea nitrogen; CICU = cardiac intensive care unit; CKD = chronic kidney disease; CRRT = continuous renal replacement therapy; CS = cardiogenic shock; GCS = glasgow coma scale; HF = heart failure; HR = heart rate; IABP = intra-aortic balloon pump; LOS = length of stay; MAP = mean arterial pressure; PAC = pulmonary arterial catheter; PCI = percutaneous intervention; RBC = red blood cell; SOFA = sequential organ failure assessment; SVT = supraventricular tachycardia; VF = ventricular fibrillation; VT = ventricular tachycardia; WBC = white blood cell.

### Admission chloride and hospital mortality by admission diagnosis

**Fig 2:**
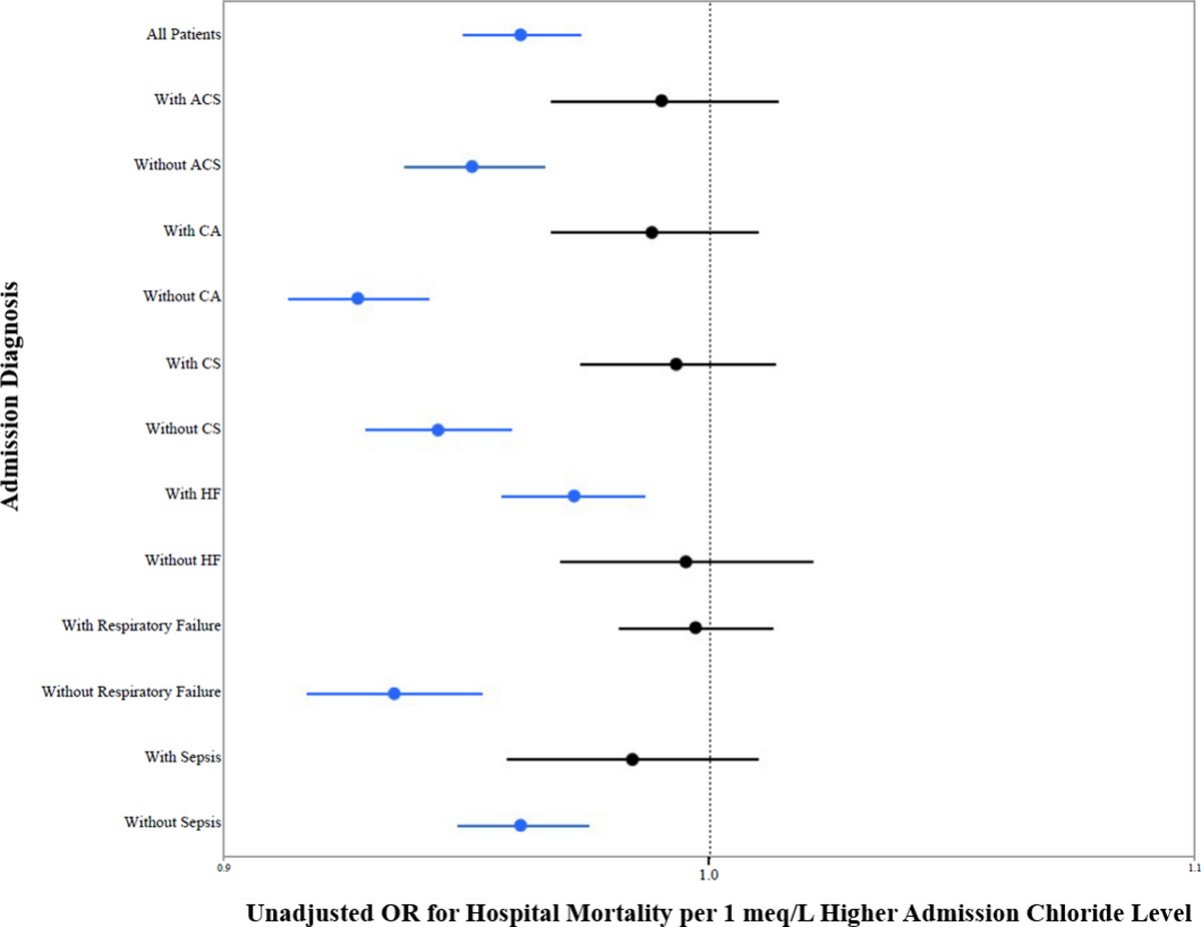
Hospital mortality and admission chloride level according to admission diagnosis.

We examined the association between admission chloride and unadjusted in-hospital mortality in patients according to admission diagnosis. We saw similar U-shaped relationships between admission chloride and in-hospital mortality demonstrated for the overall population. When we examined chloride as a continuous variable in patients with and without each of these diagnoses, we found that chloride was associated with mortality only in patients with HF, patients without ACS, and patients without critical care diagnoses **(Fig 2)**.

### Other electrolytes and hospital mortality

**Fig 3:**
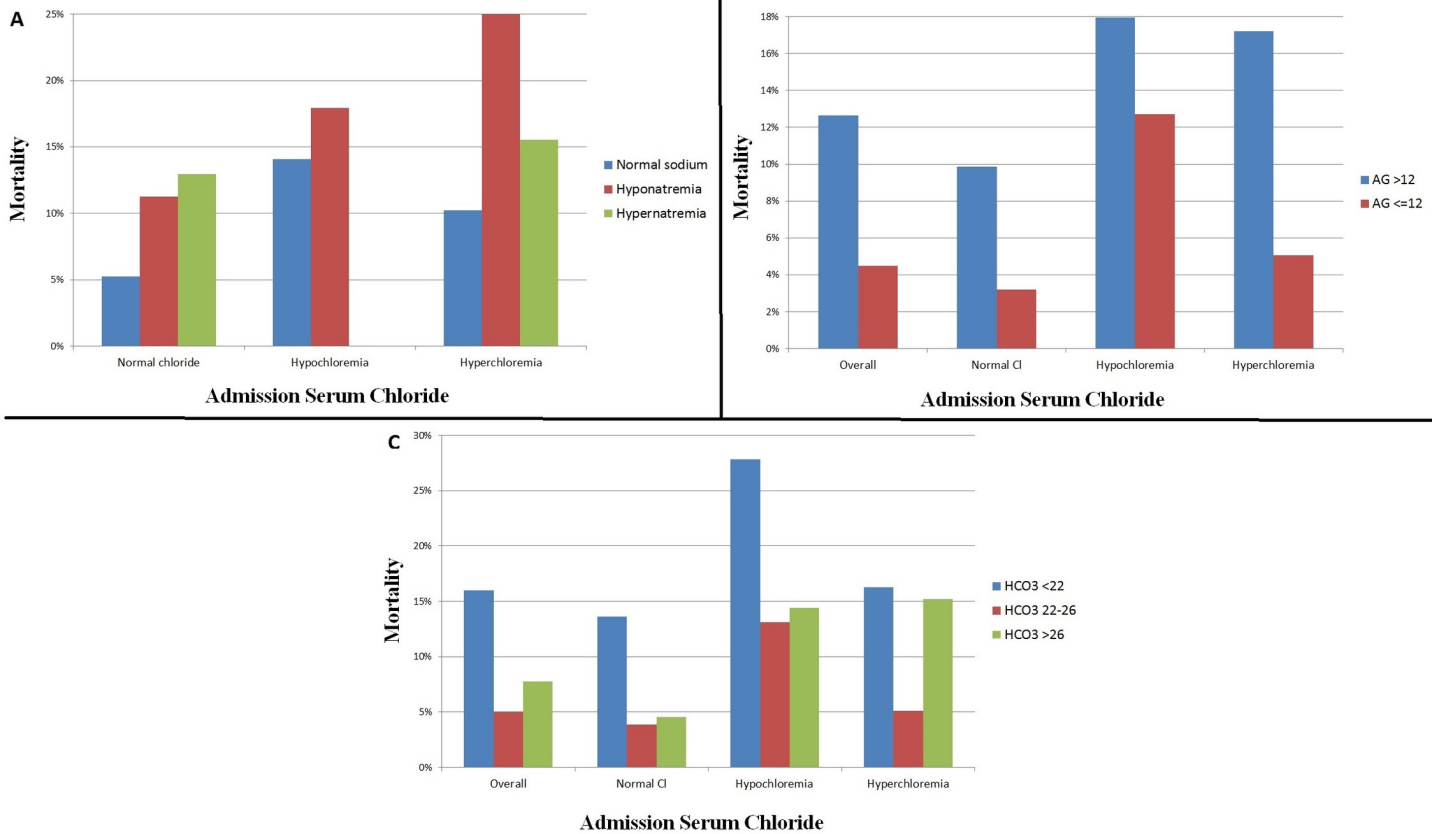
Hospital mortality and admission chloride level in the overall population, based on the admission sodium (A), admission anion gap (B), and acid-base status (C). Hypochloremia is defined as chloride <98 mEq/L and hyperchloremia is defined as chloride ≥108 mEq/L. Hyponatremia is defined as sodium <135 and hypernatremia is defined as sodium ≥145. Metabolic acidosis is defined as bicarbonate <22 and metabolic alkalosis is defined as bicarbonate >26.

A positive correlation was observed between admission chloride and sodium (r = 0.645, p <0.001), with abnormal serum sodium levels being common in patients with either hypochloremia or hyperchloremia (**Table 1**). The relationship between admission chloride and sodium levels with hospital mortality is shown in **Fig 3A** (p<0.001 for across all groups).

A negative correlation was seen between admission chloride level and anion gap (r = -0.425, p <0.001). An elevated anion gap was present in 3022 (32.1%) patients (**Table 1**). Patients with an elevated admission anion gap >12 mEq/L had higher overall mortality than those with an anion gap ≤12 mEq/L at all levels of admission chloride (p<0.001, **Fig 3B**).

A negative correlation was seen between admission chloride and bicarbonate levels (r = -0.356, p <0.001). Metabolic acidosis was present in 2,429 (25.8%) patients, and metabolic alkalosis was present in 2,218 (23.5%) patients (**Table 1**). The hospital mortality varied as a function of admission bicarbonate and admission chloride levels (p<0.001, **Fig 3C**).

### Maximum and minimum chloride

**Fig 4:**
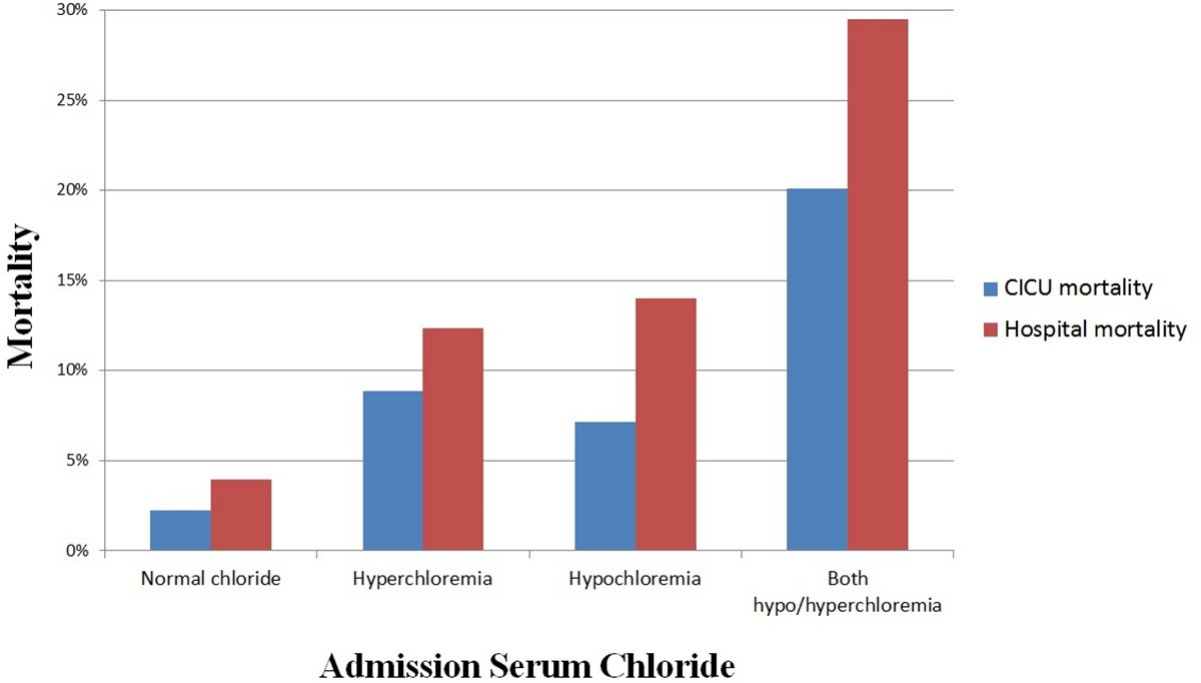
CICU and hospital mortality during hospitalization as a function of hypochloremia and hyperchloremia during the CICU stay. Hypochloremia is defined as chloride <98 mEq/L and hyperchloremia is defined as chloride ≥108 mEq/L.

Hypochloremia during the CICU stay was present in 1,980 (21.0%) patients, while hyperchloremia was present in 2,595 (27.5%) patients, including 149 (1.5%) patients with both hypochloremia and hyperchloremia during their CICU stay. Among patients with normal admission chloride, 8.8% developed hypochloremia, and 14.2% developed hyperchloremia, including 0.7% who developed both. Incremental increases in CICU and hospital mortality (**Fig 4**) were seen in patients with hyperchloremia, hypochloremia or both during CICU admission, compared with patients having normal CICU chloride levels. U-shaped relationships were observed between both minimum and maximum CICU chloride levels and CICU and in-hospital mortality (**S3A and S3B Fig**). The range of chloride variation (maximum minus minimum) during CICU stay was positively associated with hospital mortality (unadjusted OR 1.12, 95% CI 1.10–1.13, p <0.001). Hospital mortality was higher among patients with increasing chloride (in-hospital mortality 10.6%, unadjusted OR 1.656, 95% CI 1.36–2.02, p<0.001) or decreasing chloride (in-hospital mortality 8.2%, unadjusted OR 1.25, 95% CI 1.03–1.51, p = 0.023) compared with patients without a change in chloride (in-hospital mortality 6.7%).

### Post-discharge mortality

**Fig 5:**
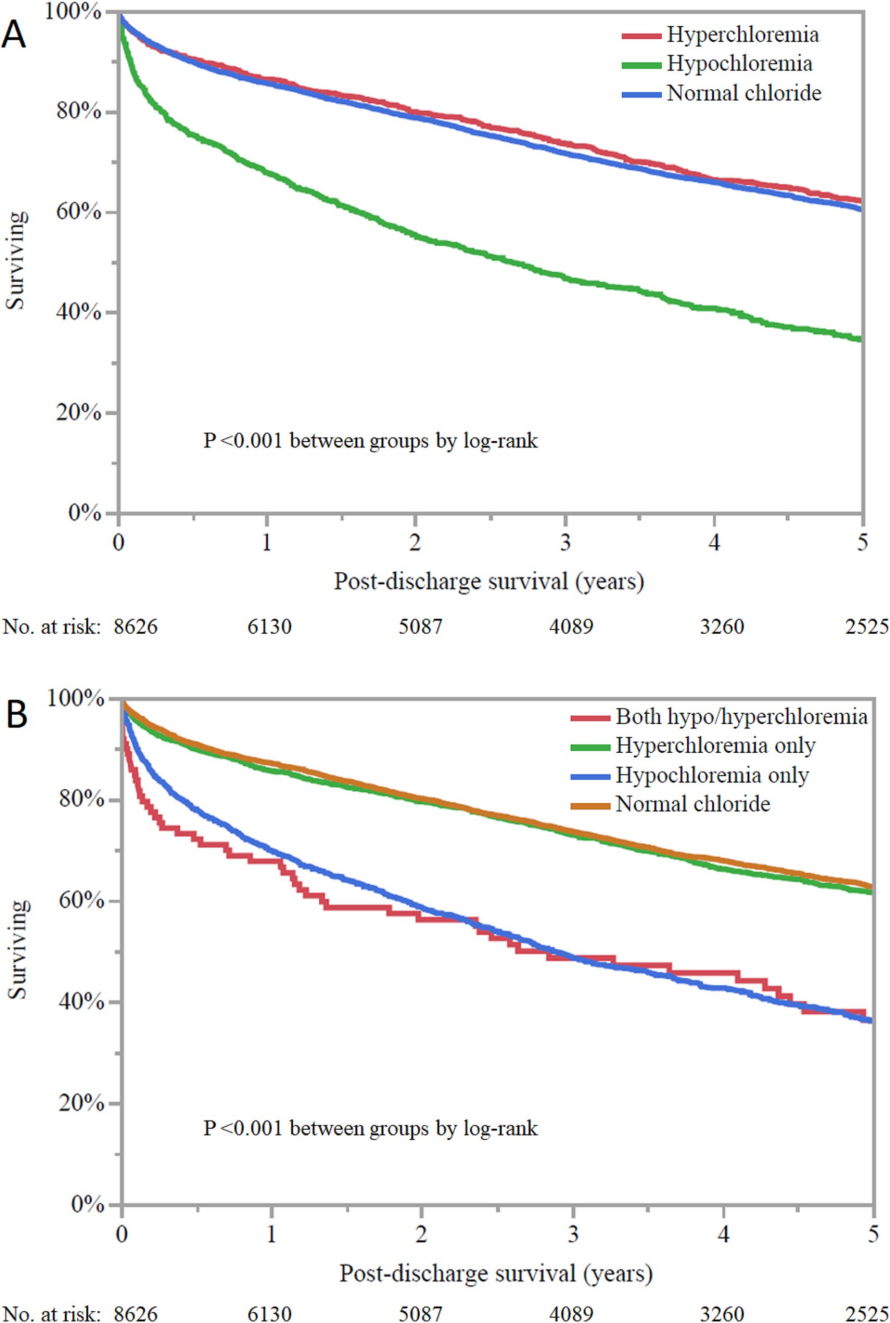
Kaplan-Meier curves demonstrating post-discharge survival among hospital survivor as a function of admission chloride (A) and minimum and maximum chloride during CICU stay (B). Hypochloremia is defined as chloride <98 mEq/L and hyperchloremia is defined as chloride ≥108 mEq/L.

A total of 2,862 (33.2%) out of 8,626 hospital survivors died during follow-up, with a mean survival of 3.4±2.9 years; 1,200 (13.0%) patients had less than 1 year of follow-up after discharge. On Kaplan-Meier analysis (**Fig 5A**), hospital survivors with admission hypochloremia had lower unadjusted post-discharge survival than patients with either admission hyperchloremia or normal admission chloride (p <0.001 by log-rank); post-discharge survival was not different for patients with admission hyperchloremia compared with normal admission chloride levels. The admission chloride level was inversely associated with post-discharge mortality (adjusted HR 0.97, 95% CI 0.96–0.98, p <0.001; **Table 2**). Hospital survivors with admission hypochloremia had higher adjusted post-discharge mortality than patients with either admission hyperchloremia (adjusted HR 1.25, 95% CI 1.10–1.43, p <0.001) or normal admission chloride (adjusted HR 1.32, 95% CI 1.08–1.60; p <0.001); post-discharge survival was not different for patients with admission hyperchloremia compared with a normal chloride level (p = 0.46).

Hospital survivors with hypochloremia during the CICU stay had lower unadjusted post-discharge survival (p <0.001 by log-rank), regardless of the presence or absence of hyperchloremia during the CICU stay; patients with isolated hyperchloremia had similar survival compared with patients having normal CICU chloride (**Fig 5B**). After adjustment, hospital survivors with isolated hypochloremia during the CICU stay had higher adjusted post-discharge mortality compared with patients with normal chloride (adjusted HR 1.26, 95% CI 1.12–1.41, p <0.001) or isolated hyperchloremia (adjusted HR 1.33, 95% CI 1.14–1.56, p <0.001). The presence of hyperchloremia was not associated with mortality compared to patients with normal chloride or concomitant hypochloremia.

## Discussion

This is the first study examining the association between chloride levels with short- and long-term mortality in a contemporary CICU population, highlighting the importance of serum chloride abnormalities as a novel prognostic factor in CICU patients. In this retrospective single-center cohort of 9,426 unique patients, we saw a U-shaped relationship between serum chloride and crude in-hospital mortality. However, lower chloride was associated with higher mortality on logistic regression, and only hypochloremia was associated with in-hospital mortality after multivariable adjustment for relevant covariates. The association between hypochloremia and mortality persisted after adjustment for other electrolytes and acid-base status, although we observed notable variability in the mortality associated with abnormal chloride levels in patients with concomitant sodium and acid-base abnormalities.

There are numerous mechanisms that can lead to the development of abnormal serum chloride in the CICU population. Chloride is the most abundant anion in the plasma and extracellular fluid and is mainly regulated via the gastrointestinal and renal systems; as with sodium levels, chloride levels will vary with plasma tonicity reflecting solute and water balance [9, 12]. Chloride levels are influenced by the accumulation of organic and nonorganic anions, and accumulation of bicarbonate (metabolic alkalosis) or organic acids (anion gap acidosis) in the plasma can contribute to hypochloremia. Impaired reabsorption of chloride within the gastrointestinal system caused by vomiting or diarrhea can result in hypochloremia (typically with associated metabolic alkalosis) [31]. Abnormal regulation of chloride balance by the kidney likely drives dyschloremia among patients with HF (who accounted for the majority of patients with hypochloremia), as decreased effective circulating volume leads to the neurohormonal secretion of aldosterone and antidiuretic hormone, resulting in increased water reabsorption with dilutional hypochloremia and hyponatremia. Furthermore, loop and thiazide diuretics used for management of hypervolemia in HF patients can disrupt renal chloride reabsorption and lead to increased chloride excretion and concomitant hypochloremic metabolic alkalosis that can be aggravated by volume contraction [32]. An elevated anion gap acidosis (often due to lactic acidosis) has been identified as an important predictor of adverse outcomes in CICU patients and likely contributes to hypochloremia in patients without metabolic alkalosis [13]. Chloride levels can be further influenced by iatrogenic resuscitation with intravenous fluids, and administration chloride-rich 0.9% normal saline has been shown to lead to the development of hyperchloremic metabolic acidosis and worse outcomes in surgical patients [33, 34].

Among patients with admission hyperchloremia, we observed an elevated risk of in-hospital mortality in our unadjusted analyses. Prior studies have similarly demonstrated an elevated risk of short-term mortality among critically ill patients with hyperchloremia, as well as patients who develop worsening hyperchloremia within 48 to 72 hours of admission [1, 35, 36]. However, in our study of unselected CICU patients, hyperchloremia was not associated with in-hospital mortality after multivariable adjustment, suggesting that the association between hyperchloremia and mortality may have been due to higher overall illness severity rather than an independent effect.

Hypochloremia was a more important prognostic indicator in our study and was correlated with increased in-hospital and post-discharge mortality after multivariable adjustment. Hypochloremia is prevalent among patients with HF due to use of diuretics and excess water retention, and most patients in our study with hypochloremia had HF. Hypochloremia is also frequently seen among critically ill patients, with incidences as high as 37% upon admission [37]. Prior studies have shown an association between hypochloremia with increased mortality and higher APACHE II scores among ICU patients [2]. Hypochloremia was recently identified as an independent risk factor for hospital mortality among critically ill septic patients when adjusted for other electrolyte disturbances [3]. While we observed a U-shaped relationship with crude hospital mortality, lower chloride was associated with higher mortality on logistic regression, and only hypochloremia was associated with mortality after multivariable adjustment.

Despite these prior studies, the impact of serum chloride is often overshadowed by other electrolytes, namely sodium. Hyponatremia has long been established as an important prognostic variable among patients with HF, as well as other patients with acute cardiovascular disease [38–40]. As with hyponatremia, hypochloremia is common in patients with underlying HF, with incidences ranging from 13–23% [12]. Serum chloride has been demonstrated as an independent predictor of long-term mortality among patients with decompensated systolic HF, while this association has not been true for serum sodium when adjusted for chloride [5]. These findings have been replicated in several studies of short- and long-term mortality in patients with acute decompensated and chronic HF [6–8]. Our study in unselected CICU patients replicates these findings by demonstrating that hypochloremia was associated with higher in-hospital and post-discharge mortality, even when adjusting for other electrolyte disturbances. Therefore, chloride levels may provide a more robust predictive value than sodium levels by integrating the effects of plasma tonicity (as reflected by serum sodium) with the impact of acid-base homeostasis.

As with abnormal sodium levels, it remains uncertain whether abnormal chloride levels are directly pathogenic or merely a marker of sicker, high-risk patients. Patients with admission hypochloremia had higher illness severity than the rest of our population, with a greater comorbidity burden and more heart, respiratory and renal failure. Thus, hypochloremia may be simply a marker of elevated mortality risk, which would also explain why a low admission chloride level on CICU admission was predictive of a higher mortality at 5 years. However, dyschloremia (particularly hypochloremia) has been linked to several pathogenic mechanisms that could plausibly contribute directly to adverse outcomes in this group, including disruption of acid-base and fluid balance.

Because the pathologic role of chloride is poorly understood, it is unclear why hypochloremia was an independent predictor of poor outcomes while hyperchloremia unexpectedly was not. This may relate to the interplay between serum chloride and acid-base homeostasis, whereby either anion gap metabolic acidosis or hypochloremic metabolic alkalosis can contribute simultaneously to low chloride levels and worse outcomes. However, hypochloremia was an independent predictor of mortality among unselected CICU patients independent of acid-base status, and the majority of these patients had a concomitant metabolic alkalosis. Alkalosis has been associated with poorer outcomes among critically ill populations and could have contributed to the increased mortality rates seen in our hypochloremic patients [41]. However, the subset of patients in our study with the poorest outcomes were those with an elevated anion gap and metabolic acidosis, especially in the presence of hypochloremia. Hypochloremia may result from anion-gap acidosis, which has been associated with increased mortality among critically ill patients, including CICU patients [13]. While hyperchloremia was not associated with adjusted mortality, it is notable that unadjusted mortality was particularly high among hyperchloremic patients with an elevated anion gap despite no association between hyperchloremia and adjusted mortality. The majority of hyperchloremic patients had a concomitant metabolic acidosis, and the presence of concomitant hyperchloremic acidosis and anion gap acidosis could have led to greater severity of acidosis and less favorable outcomes.

The correlation between hypochloremia and adverse outcomes could also relate to the lower plasma tonicity in hypochloremic patients; similarly, hyponatremia was associated with higher adjusted mortality in a prior study from this CICU cohort but hypernatremia was not [27]. Chloride directly influences the renal reabsorption of sodium, intrarenal vasoregulatory mechanisms, and the renin-angiotensin-aldosterone system regulation [42, 43]. This is particularly important among patients with underlying HF, as low baseline chloride has been linked to decreased responses to diuretic therapy [6, 7]. These numerous underlying physiologic effects of chloride imply that hypochloremia may have direct pathogenic effects on fluid status, renal function, and acid-base balance in CICU patients that may not be observed with hyperchloremia.

While our study shows that hypochloremia is a novel adverse prognostic factor among CICU patients, it is unclear whether correction of underlying chloride abnormalities would affect in-hospital or post-discharge mortality. This is particularly salient considering the numerous potential ways in which chloride levels can change in response to various therapies. An increase in serum chloride concentration within 24 hours was shown to correlate with improved survival among hypochloremic patients with severe sepsis or septic shock, suggesting the potential for therapeutic interventions affecting serum chloride to improve outcomes [3]. However, in our population, the variation in serum chloride levels during CICU was associated with increased unadjusted mortality and patients with hypochloremia and hyperchloremia during the CICU admission did the worst. We do not have data regarding the etiology of these chloride variations, and they may reflect a more complicated CICU stay involving greater fluid and electrolyte shifts. The effect of chloride variations on in-hospital mortality may be due to greater underlying illness severity as opposed to direct pathologic effects of dyschloremia. Future studies should analyze the effect of strategies targeting serum chloride levels on outcomes in CICU patients.

### Limitations

As with all historical cohort studies, this study cannot be used to determine causal relationships and unmeasured confounding variables may have mediated correlation between chloride levels and mortality. Specifically, patients with hypochloremia were substantially sicker than other patients and it is possible that the observed association between hypochloremia and adverse outcomes is due to residual confounding despite multivariable adjustment. This was a single-center study, and our CICU practice may differ from other medical facilities or regions. We could not obtain data on the use of diuretics, type of resuscitation fluids used, or overall fluid balance, which are all known to influence chloride levels; this limits our ability to infer the specific causes of dyschloremia. As such, we are unable to clearly delineate the etiology of the abnormal chloride levels to determine whether any specific cause of dyschloremia was associated with worse outcomes. The ICD-9 admission diagnoses we obtained reflect all diagnoses and cannot distinguish the primary admission diagnosis.

## Conclusion

Our study demonstrated a U-shaped relationship between admission chloride levels and unadjusted hospital and CICU mortality. After multivariate adjustment, hypochloremia was associated with in-hospital and post-discharge mortality, independent of other electrolytes, including sodium. Hypochloremia remained an important predictor of mortality in patients with concomitant abnormalities of sodium and acid-base balance. Our data highlights the importance of serum chloride abnormalities in the CICU population and suggests an important role of disorders affecting chloride homeostasis among patients with cardiovascular disease. Future studies are needed to determine the importance of chloride within different cardiovascular sub-populations, to establish the etiology of the underlying dyschloremia, and to determine whether correction of underlying chloride abnormalities will lead to improved patient outcomes.

## Supporting information

S1 Fig Flow diagram demonstrating inclusion/exclusion criteria for the final study population. (DOCX)

S2 Fig Histogram illustrating distribution of admission chloride levels. (DOCX)

S3 Fig CICU and hospital mortality as a function of minimum (A) and maximum (B) chloride level during the CICU stay.
(DOCX)

S1 Data (XLSX)
