# Supplementary material for: Abnormal serum chloride is associated with increased mortality among unselected cardiac intensive care unit patients
Source: PLoS One. 2021 Apr 26;16(4):e0250292. doi: 10.1371/journal.pone.0250292 (PMC8075550; doi:10.1371/journal.pone.0250292)
Supplement: S1 Fig — (DOCX) [file pone.0250292.s001.docx]

**S1 Fig:** Flow diagram demonstrating inclusion/exclusion criteria for the final study population.

**
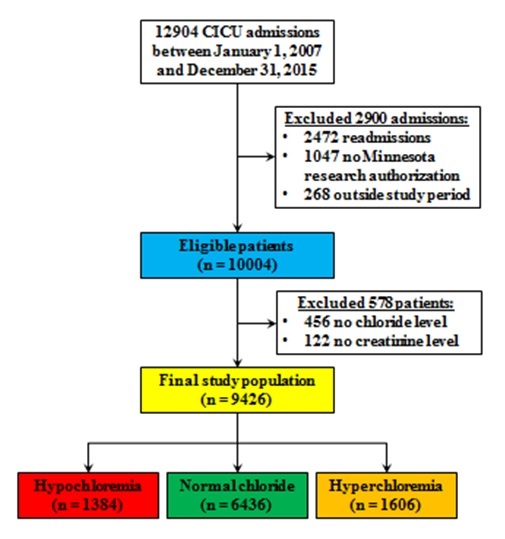
**
