# Supplementary figures and images for: Abnormal serum chloride is associated with increased mortality among unselected cardiac intensive care unit patients
Source: PLoS One. 2021 Apr 26;16(4):e0250292. doi: 10.1371/journal.pone.0250292 (PMC8075550; doi:10.1371/journal.pone.0250292)

**S2 Fig:** Histogram illustrating distribution of admission chloride levels.


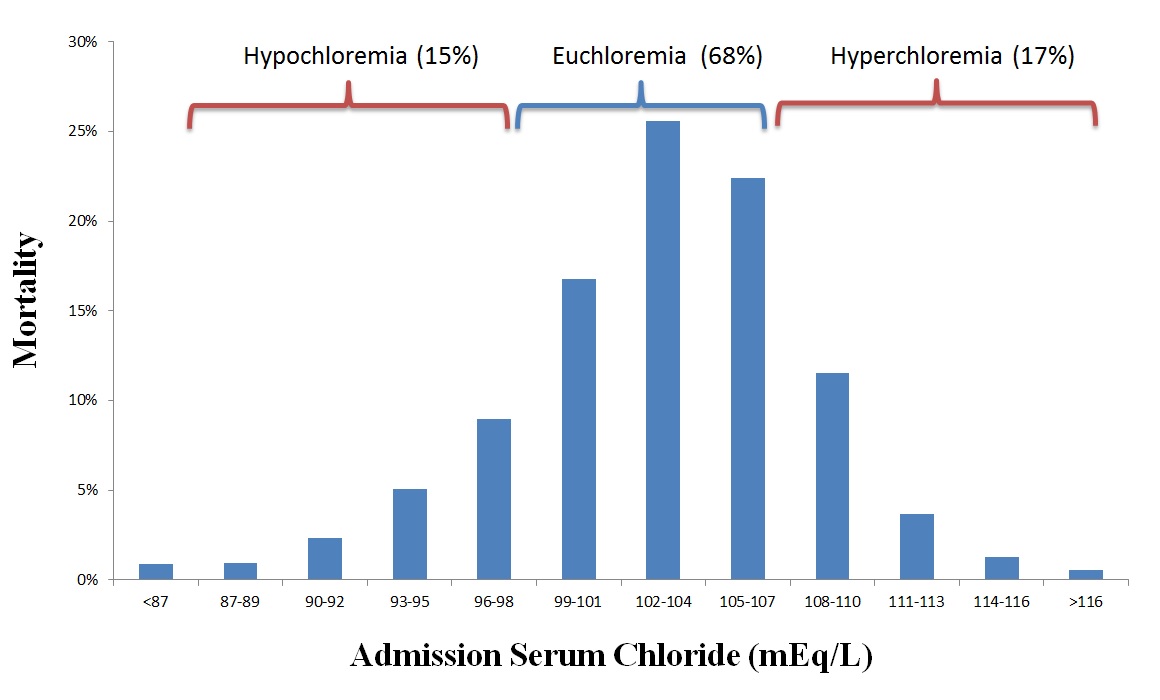

Supplement: S2 Fig — (DOCX) [file pone.0250292.s002.docx]
