# Supplementary material for: Abnormal serum chloride is associated with increased mortality among unselected cardiac intensive care unit patients
Source: PLoS One. 2021 Apr 26;16(4):e0250292. doi: 10.1371/journal.pone.0250292 (PMC8075550; doi:10.1371/journal.pone.0250292)

**S3 Fig:** CICU and hospital mortality as a function of minimum (A) and maximum (B) chloride level during the CICU stay.


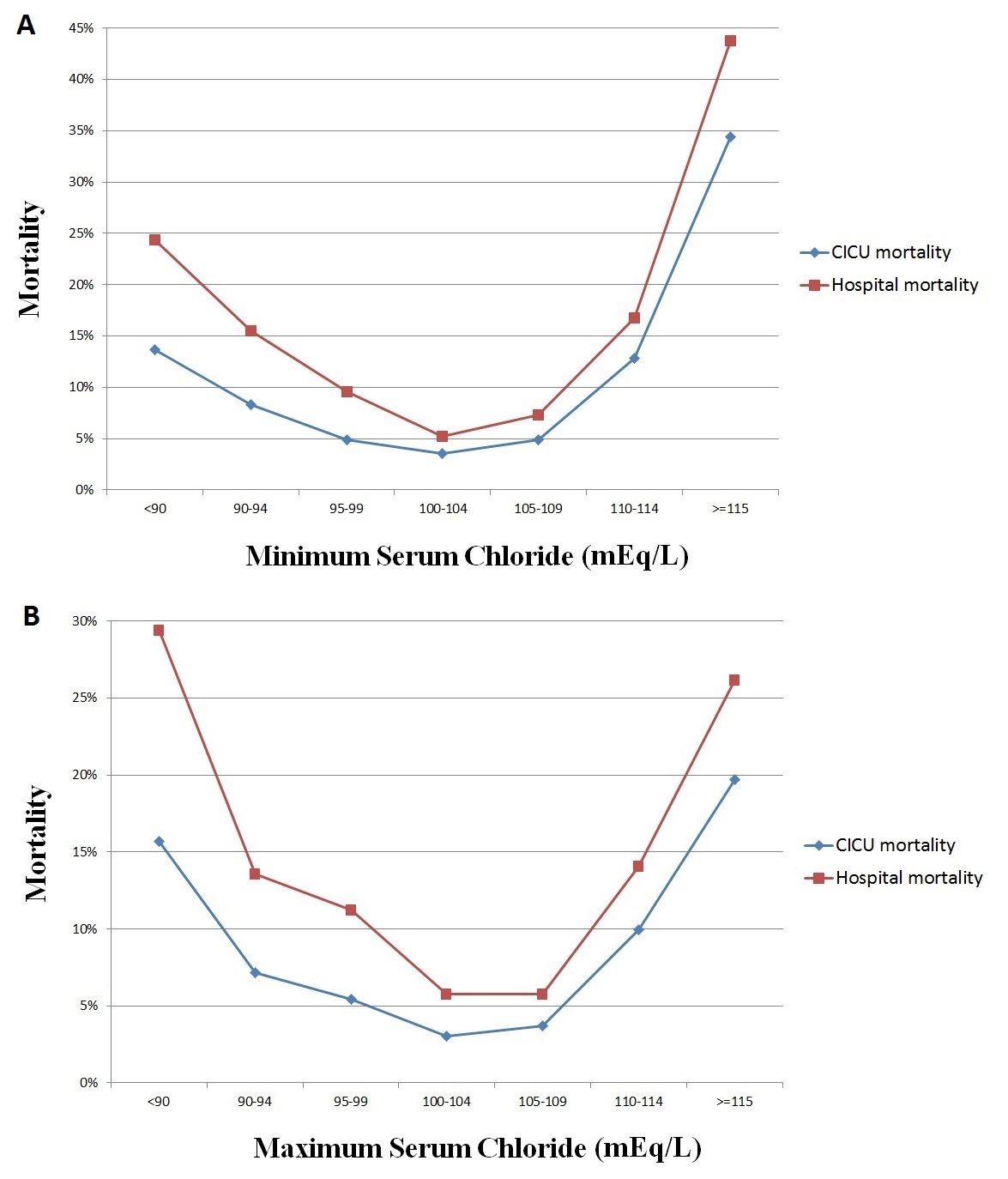

Supplement: S3 Fig — CICU and hospital mortality as a function of minimum (A) and maximum (B) chloride level during the CICU stay. (DOCX) [file pone.0250292.s003.docx]
